# Inertial forward–backward methods for solving vector optimization problems

**DOI:** 10.1080/02331934.2018.1440553

**Published:** 2018-02-20

**Authors:** Radu Ioan Boţ, Sorin-Mihai Grad

**Affiliations:** a Faculty of Mathematics, University of Vienna, Vienna, Austria.; b Faculty of Mathematics and Computer Science, Babeş-Bolyai University, Cluj-Napoca, Romania.; c Faculty of Mathematics and Computer Science, University of Leipzig, Leipzig, Germany.; d Faculty of Mathematics, Chemnitz University of Technology, Chemnitz, Germany.

**Keywords:** Vector optimization problems, inertial proximal algorithms, forward–backward algorithms, weakly efficient solutions

## Abstract

We propose two forward–backward proximal point type algorithms with inertial/memory effects for determining weakly efficient solutions to a vector optimization problem consisting in vector-minimizing with respect to a given closed convex pointed cone the sum of a proper cone-convex vector function with a cone-convex differentiable one, both mapping from a Hilbert space to a Banach one. Inexact versions of the algorithms, more suitable for implementation, are provided as well, while as a byproduct one can also derive a forward–backward method for solving the mentioned problem. Numerical experiments with the proposed methods are carried out in the context of solving a portfolio optimization problem.

## 1. Introduction and preliminaries

With this paper we propose the first, to the best of our knowledge, iterative methods for determining weakly efficient solutions to vector optimization problems consisting in vector-minimizing with respect to a convex cone the sum of two vector functions. The numerical methods we propose rely on the classical proximal point algorithm due to Martinet extended for vector optimization problems by Bonnel, Iusem and Svaiter. In order to treat the involved functions separately, we propose a forward–backward splitting scheme, to which inertial/memory effects are added. The inertial methods with memory effects were inspired from heavy ball with friction dynamical systems and have as a characteristic feature the fact that an iteration variable depends on the previous two elements of the same sequence, not only on its predecessor as it is usually the case for many algorithmic approaches. The first inertial proximal point type algorithm has been proposed by Alvarez and Attouch (cf. [1,2]) for the minimization of a proper, convex and lower semicontinuous function. To the best of our knowledge, the only other inertial type proximal method for solving vector optimization problems proposed so far in the literature is the one in [3], which follows a different path than our contribution as it is employed for determining ideally efficient solutions. The vector optimization problems we solve with our methods consist in vector-minimizing with respect to a given closed convex pointed cone the sum of a proper cone-convex vector function, evaluated in a backward step, with a cone-convex differentiable one that is evaluated in a forward step, both mapping from a Hilbert space to a Banach one. The usual way to approach vector optimization problems is by scalarizing them, but this can often lead to unbounded problems (see, for instance [4, Remark 1]), hence the need to address the vector optimization problems directly, especially when it comes to numerically solving them. One can find some results on the choice of scalarizing parameters in order to guarantee the existence of optimal solutions of the scalarized problems in the literature, but the imposed conditions are quite restrictive (see [5,6]) and their verification may prove to be too expensive from a computational point of view. This has motivated research on iterative methods for directly solving multiobjective or vector optimization problems consisting in vector-minimizing a vector function, sometimes subject to geometric constraints, and in the recent literature several contributions in this sense can be found in both smooth (cf. [7,8]) and convex nonsmooth (cf. [4,9]) cases. Different to these works, in this paper we vector-minimize with respect to a convex cone the sum of two cone-convex vector functions, that are handled separately in each iteration via a forward–backward scheme, covering thus a larger category of problems than in the existing literature. Moreover, we added inertial/memory effects to the proposed scheme that have a positive contribution to its robustness and speed. The proximal point scheme proposed in [4] is recovered as a special case of our method when the inertial steps and the smooth objective term are removed. Moreover, unlike the mentioned papers where iterative methods for solving vector optimization problems were proposed, but their implementation was left for later due to the difficulty of solving the employed subproblems, we present a concrete application as well that is solved in Matlab.

For implementation purposes, we provide also versions of the inertial forward–backward algorithms which do not scalarize the original problem, but some approximations of it, another one at each iteration, as done for instance also in [4,9]. We have opted for the linear scalarization of the intermediate vector optimization problems instead of other existing alternatives (see, for instance, [10, Section 4.4]) for both simplicity and computational reasons. The construction of the algorithms guarantees the existence of an optimal solution to each of the considered (linearly) scalarized optimization problems, a feature usually mentioned as an advantage of other scalarization techniques in comparison to the linear one. Moreover, the linear scalarization is more flexible than its counterparts, allowing modifications of its parameters at each iteration.

Let *X* be a Hilbert space and *Y* a separable Banach space that is partially ordered by a pointed closed convex cone C⊆Y. The partial ordering induced by *C* on *Y* is denoted by ‘${≦}_{C}$’ (i.e. it holds $x{≦}_{C}y$ when y-x∈C, where x,y∈Y) and we write $x\leq_{C}y$ if $x{≦}_{C}y$ and x≠y. A greatest element with respect to ‘${≦}_{C}$’ denoted by $\infty_{C}$ which does not belong to *Y* is attached to this space, and let $Y^{∙}=Y\cup \left\{\infty_{C}\right\}$. Then for any y∈Y one has $y\leq_{C}\infty_{C}$ and we consider on $Y^{∙}$ the operations $y+\infty_{C}=\infty_{C}+y=\infty_{C}$ for all $y\in Y^{∙}$ and $t\cdot \infty_{C}=\infty_{C}$ for all t≥0. By $\left\langle y^{*},y\right\rangle$ we denote the value at y∈Y of the linear continuous functional $y^{*}\in Y^{*}$ and by convention we take $\langle y^{*},\infty_{C}\rangle =+\infty$ for all $y^{*}\in C^{*}$, where $C^{*}=\{y^{*}\in Y^{*}:\left\langle y^{*},y\right\rangle \geq 0$∀y∈C} is the *dual cone* to *C*. Given a subset *U* of *X*, by clU, intU and $\delta_{U}$ we denote its *closure*, *interior* and *indicator function*, respectively.

When $f:X\to \bar{R}=R\cup \left\{\pm \infty \right\}$ is *proper* (i.e. is nowhere equal to -∞ and has at least a real value) and ε≥0, if f(x)∈R the *(convex) ε-subdifferential* of *f* at *x* is $\partial_{\varepsilon }f\left(x\right)=\{x^{*}\in X^{*}:f\left(y\right)-f\left(x\right)\geq \left\langle x^{*},y-x\right\rangle -\varepsilon$∀y∈X}, while if f(x)=+∞ we take by convention $\partial_{\varepsilon }f\left(x\right)=\emptyset$. The ε-subdifferential of *f* becomes in case ε=0 its classical *(convex) subdifferential* denoted by ∂f. Then $\overset{¯}{x}\in X$ is a minimum of *f* if and only if $0\in \partial f(\overset{¯}{x})$. Denote also by ${\left[t\right]}_{+}=\max\left\{t,0\right\}$ for any t∈R.

A vector function $F:X\to Y^{∙}=Y\cup \left\{\infty_{C}\right\}$ is said to be *proper* if its *domain*domF={x∈X:F(x)∈Y} is nonempty, *C-convex* if $F\left(tx+\left(1-t\right)y\right){≦}_{C}tF\left(x\right)+\left(1-t\right)F\left(y\right)$ for all x,y∈X and all t∈[0,1] and *positively C-lower semicontinuous* (in the literature also *star *C*-lower semicontinuous*) when the function $x\mapsto \langle z^{*},F\left(x\right)\rangle$, further denoted by $\left(z^{*}F\right):X\to \bar{R}$, is lower semicontinuous for all $z^{*}\in C^{*}\backslash \left\{0\right\}$.

Assume further that intC≠∅. Consider the vector optimization problem

$$\left(VP\right)\;\;\underset{x\in X}{\;\text{WMin}}[F\left(x\right)+G\left(x\right)],$$

where F:X→Y is a Fréchet differentiable vector function with an *L*-Lipschitz continuous gradient ∇F and $G:X\to Y^{∙}$ is a proper vector function. It is the aim of this paper to provide a proximal inertial forward–backward algorithm for determining the weakly efficient solutions to (*VP*). An element $\overset{¯}{x}\in \;\text{dom}G$ is said to be an *efficient solution* to (*VP*) if there is no x∈X such that $F\left(x\right)+G\left(x\right)\leq_{C}F\left(\overset{¯}{x}\right)+G\left(\overset{¯}{x}\right)$ and a *weakly efficient solution* to (*VP*) if $(F(\overset{¯}{x})+G(\overset{¯}{x})-\;\text{int}C)\cap (F+G)(\;\text{dom}G)=\emptyset$, respectively. We denote by E(VP) the set of all efficient solutions to (*VP*) and by WE(VP) the one of all weakly efficient ones. From [10, Corollary 2.4.26] one has the following characterization of the weakly efficient solutions to (*VP*) by means of a linear scalarization.

Lemma 1.1: If F+G is *C*-convex, then $\overset{¯}{x}\in \mathrm{WE}(VP)$ if and only if

$$\exists z^{*}\in C^{*}\backslash \left\{0\right\}:\;\left\langle z^{*},F\left(\overset{¯}{x}\right)+G\left(\overset{¯}{x}\right)\right\rangle \leq \left\langle z^{*},F\left(x\right)+G\left(x\right)\right\rangle \;\forall x\in X.$$

Remark 1: A sufficient hypothesis for guaranteeing the *C*-convexity of F+G is to take both *F* and *G* to be *C*-convex.

Lemma 1.2: When *F* is *C*-convex, then for any $z^{*}\in C^{*}$ the function $(z^{*}F):Y\to R$ is convex and Fréchet differentiable with an $L\|z^{*}\|$-Lipschitz continuous gradient.

**Proof** Let $z^{*}\in C^{*}$. The convexity and continuous differentiability of $\left(z^{*}F\right)$ were already proven in the literature, so we focus on the Lipschitz continuity of its gradient, that, by the chain rule, coincides at any x∈X with the functional $z^{*}\circ \nabla F\left(x\right)$ defined by $w\in X\mapsto \langle z^{*},\nabla F\left(x\right)\left(w\right)\rangle$.

For x,y∈X, one has, keeping in mind the linearity of $z^{*}$ and of the gradient,

$$\begin{array}{cc} \|\nabla \left(z^{*}F\right)\left(x\right)-\nabla \left(z^{*}F\right)\left(y\right)\| & =\|z^{*}\circ \nabla F\left(x\right)-z^{*}\circ \nabla F\left(y\right)\|=\|z^{*}\circ \left(\nabla F\left(x\right)-\nabla F\left(y\right)\right)\|\\ & \leq \|z^{*}\left\|\|\nabla F\left(x\right)-\nabla F\left(y\right)\|\leq L\right\|z^{*}\|\|x-y\|, \end{array}$$

therefore the gradient of $\left(z^{*}F\right)$ is $L\|z^{*}\|$-Lipschitz continuous.

A result which is very useful in proving the convergence of numerical algorithms is the celebrated Opial’s Lemma (cf. [11]).

Lemma 1.3: Let ${\left(x_{n}\right)}_{n}\subseteq X$ be a sequence and S⊆X a nonempty set such that

(a) $\lim_{n\to +\infty }\left\|x_{n}-x\right\|$ exists for every x∈S;
(b) if $x_{n_{j}}⇀z$ for a subsequence $n_{j}\to +\infty$, then z∈S,

where ‘⇀’ denotes the convergence in the weak topology. Then, there exists an $\overset{¯}{x}\in S$ such that $x_{n}⇀\overset{¯}{x}$ when n→+∞.

## 2. Inertial forward–backward algorithm

We propose below an exact proximal inertial forward–backward iterative method for determining the weakly efficient solutions to (*VP*). It generates a sequence ${\left(x_{n}\right)}_{n}\subseteq X$ that, as seen later, converges under suitable but not very demanding hypotheses towards a weakly efficient solution to (*VP*).


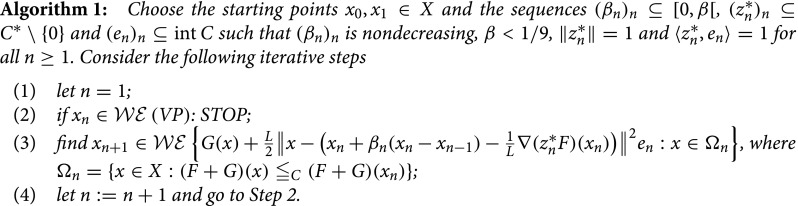

Remark 2: When F≡0, Algorithm 1 becomes an inertial proximal point method for solving vector optimization problems, which by additionally taking $\beta_{n}=0$ for all n≥1 collapses into the proximal point method for vector-minimizing a nonsmooth vector function introduced in [4]. On the other hand, when Y=R and $C=R_{+}$ (i.e. in the scalar case), Algorithm 1 becomes the inertial proximal-gradient method for scalar optimization problems, that can be derived from the algorithm for finding zeros of maximally monotone operators proposed in [12]. When, furthermore, F≡0, it collapses into the one from [1], while when $\beta_{n}=0$ for all n≥1 it becomes the celebrated ISTA method (see, for instance, [13]), however in a more general framework.

Remark 3: Analyzing Algorithm 1 one can notice that at every iteration a different vector optimization problem is addressed, each of them having a smaller feasible set than its predecessor.

Before formulating the convergence statement concerning the sequence ${\left(x_{n}\right)}_{n}$ generated by Algorithm 1, it is necessary to introduce a new notion, considered in most of the papers dealing with proximal methods for vector optimization problems (see [4,9]).

Definition 2.1: Given $x_{0}\in X$, the set $F\left(X\right)\cap (F\left(x_{0}\right)-C)$ is said to be *C-complete* when for all sequences ${\left(a_{n}\right)}_{n}\subseteq X$ with $a_{0}=x_{0}$ such that $F\left(a_{n+1}\right){≦}_{C}F\left(a_{n}\right)$ for all n≥1 there exists an a∈X such that $F\left(a\right){≦}_{C}F\left(a_{n}\right)$ for all n≥1.

Theorem 2.1: Let *F* be *C*-convex and *G* be *C*-convex and positively *C*-lower semicontinuous and assume that $\left(F+G\right)\left(X\right)\cap (F\left(x_{0}\right)+G\left(x_{0}\right)-C)$ is *C*-complete. If Algorithm 1 does not stop in finitely many steps, then any sequence ${\left(x_{n}\right)}_{n}$ generated by it converges weakly towards a weakly efficient solution to (*VP*).

**Proof** We show first that the algorithm is well defined, i.e. if the stopping rule is not activated the next iteration exists. Assuming that we have obtained an $x_{n}$, where n≥1, we have to secure the existence of $x_{n+1}$.

The hypotheses imposed on *F* and *G* guarantee that the set $\Omega_{n}$ is convex and closed, since *G* is *C*-epi closed and *F* is Fréchet differentiable. Thus, $\delta_{\Omega_{n}}$ is convex and lower semicontinuous. As the function

$$x\mapsto \langle z_{n}^{*},G\left(x\right)+\frac{L}{2}{∥x-x_{n}-\beta_{n}\left(x_{n}-x_{n-1}\right)+\frac{1}{L}\nabla \left(z_{n}^{*}F\right)\left(x_{n}\right)∥}^{2}e_{n}\rangle +\delta_{\Omega_{n}}\left(x\right)$$

is lower semicontinuous, being a sum of lower semicontinuous and continuous functions, and strongly convex, as the sum of some convex functions and a squared norm, it has exactly one minimum. By Lemma 1.1 this minimum is a weakly efficient solution to the vector optimization problem in Step 3 of Algorithm 1 and we denote it by $x_{n+1}$.

Thus one has

$$0\in \partial \left(\langle z_{n}^{*},G\left(\cdot \right)+\frac{L}{2}{∥\cdot -x_{n}-\beta_{n}\left(x_{n}-x_{n-1}\right)+\frac{1}{L}\nabla \left(z_{n}^{*}F\right)\left(x_{n}\right)∥}^{2}e_{n}\rangle +\delta_{\Omega_{n}}\left(\cdot \right)\right)\left(x_{n+1}\right),$$

which, due to the continuity of the norm, turns into (cf. [10, Theorem 3.5.5])

$$\begin{array}{cc} 0 & \in \;\partial \left(\langle z_{n}^{*},G\left(\cdot \right)+\delta_{\Omega_{n}}\left(\cdot \right)\rangle \right)\left(x_{n+1}\right)\\ & \;+L\partial \left(\frac{1}{2}{∥\cdot -x_{n}-\beta_{n}\left(x_{n}-x_{n-1}\right)+\frac{1}{L}\nabla \left(z_{n}^{*}F\right)\left(x_{n}\right)∥}^{2}\right)\left(x_{n+1}\right). \end{array}$$

or, equivalently,

$$-L\left(x_{n+1}-x_{n}-\beta_{n}\left(x_{n}-x_{n-1}\right)+\frac{1}{L}\nabla \left(z_{n}^{*}F\right)\left(x_{n}\right)\right)\in \partial \left(\left\langle z_{n}^{*},G\left(\cdot \right)\right\rangle +\delta_{\Omega_{n}}\left(\cdot \right)\right)\left(x_{n+1}\right).$$

Thus, for any $x\in \Omega_{n}$, one has

(1) $$\begin{array}{c} \left\langle z_{n}^{*},G\left(x\right)-G\left(x_{n+1}\right)\right\rangle \geq \left\langle L\left(x_{n+1}-x_{n}-\beta_{n}\left(x_{n}-x_{n-1}\right)\right)+\nabla \left(z_{n}^{*}F\right)\left(x_{n}\right),x_{n+1}-x\right\rangle . \end{array}$$

The set $\Omega :=\cap_{k\geq 1}\Omega_{k}=\{x\in R^{n}:\left(F+G\right)\left(x\right){≦}_{C}\left(F+G\right)\left(x_{k}\right)$∀k≥1} is nonempty because of the *C*-completeness hypothesis. Let $\tilde{x}\in \Omega$. Then, using that $\tilde{x}\in \Omega_{n}$ and $z_{n}^{*}\in C^{*}$, (1) yields

$$\begin{array}{cc} 0\geq & \left\langle z_{n}^{*},\left(F+G\right)\left(\tilde{x}\right)-\left(F+G\right)\left(x_{n+1}\right)\right\rangle \\ \geq & \left\langle L\left(x_{n+1}-x_{n}-\beta_{n}\left(x_{n}-x_{n-1}\right)\right)+\nabla \left(z_{n}^{*}F\right)\left(x_{n}\right),x_{n+1}-\tilde{x}\right\rangle +\left\langle z_{n}^{*},F\left(\tilde{x}\right)-F\left(x_{n+1}\right)\right\rangle . \end{array}$$

Employing the descent lemma (e.g. [14, Theorem 18.15(iii)]) for $\left\langle z_{n}^{*},F\right\rangle$, which is convex and Fréchet differentiable with $L\|z_{n}^{*}\|=L$-Lipschitz continuous gradient, this implies

$$\left\langle L\left(x_{n+1}-x_{n}-\beta_{n}\left(x_{n}-x_{n-1}\right)\right)+\nabla \left(z_{n}^{*}F\right)\left(x_{n}\right)-\nabla \left(z_{n}^{*}F\right)\left(\tilde{x}\right),x_{n+1}-\tilde{x}\right\rangle \leq \frac{L}{2}{\left\|\tilde{x}-x_{n+1}\right\|}^{2}.$$

The $\frac{1}{L}$-cocoercivity of $\nabla (z_{n}^{*}F)$ (cf. [14, Theorem 18.15(v)]) gives

$$\begin{array}{cc} & \left\langle \nabla \left(z_{n}^{*}F\right)\left(x_{n}\right)-\nabla \left(z_{n}^{*}F\right)\left(\tilde{x}\right),x_{n+1}-\tilde{x}\right\rangle \\ & \;=\left\langle \nabla \left(z_{n}^{*}F\right)\left(x_{n}\right)-\nabla \left(z_{n}^{*}F\right)\left(\tilde{x}\right),x_{n}-\tilde{x}\right\rangle +\left\langle \nabla \left(z_{n}^{*}F\right)\left(x_{n}\right)-\nabla \left(z_{n}^{*}F\right)\left(\tilde{x}\right),x_{n+1}-x_{n}\right\rangle \\ & \;\geq \frac{1}{L}{\left\|\nabla \left(z_{n}^{*}F\right)\left(x_{n}\right)-\nabla \left(z_{n}^{*}F\right)\left(\tilde{x}\right)\right\|}^{2}+\left\langle \nabla \left(z_{n}^{*}F\right)\left(x_{n}\right)-\nabla \left(z_{n}^{*}F\right)\left(\tilde{x}\right),x_{n+1}-x_{n}\right\rangle \\ & \;=\frac{1}{L}{∥\nabla \left(z_{n}^{*}F\right)\left(x_{n}\right)-\nabla \left(z_{n}^{*}F\right)\left(\tilde{x}\right)+\frac{L}{2}\left(x_{n+1}-x_{n}\right)∥}^{2}-\frac{L}{4}{\left\|x_{n+1}-x_{n}\right\|}^{2}\\ & \;\geq -\frac{L}{4}{\left\|x_{n+1}-x_{n}\right\|}^{2}, \end{array}$$

therefore

$$\left\langle L\left(x_{n+1}-x_{n}-\beta_{n}\left(x_{n}-x_{n-1}\right)\right),x_{n+1}-\tilde{x}\right\rangle -\frac{L}{2}\|\tilde{x}-x_{n+1}{\|}^{2}-\frac{L}{4}{\left\|x_{n+1}-x_{n}\right\|}^{2}\leq 0.$$

For each k≥1 denote $\varphi_{k}=\left(1/2\right){\left\|x_{k}-\tilde{x}\right\|}^{2}$. As $\varphi_{n}-\varphi_{n+1}=\frac{1}{2}{\left\|x_{n+1}-x_{n}\right\|}^{2}+\left\langle x_{n+1}-x_{n}-\beta_{n}\left(x_{n}-x_{n-1}\right),\tilde{x}-x_{n+1}\right\rangle +\beta_{n}\left\langle x_{n}-x_{n-1},\tilde{x}-x_{n+1}\right\rangle$, the previous inequality can be rewritten, after dividing with *L*, as

$$\varphi_{n+1}-\varphi_{n}+\frac{1}{4}\|x_{n+1}-x_{n}{\|}^{2}-\frac{1}{2}{\left\|\tilde{x}-x_{n+1}\right\|}^{2}-\beta_{n}\left\langle x_{n}-x_{n-1},x_{n+1}-\tilde{x}\right\rangle \leq 0,$$

and, since $\left\langle x_{n}-x_{n-1},x_{n+1}-\tilde{x}\right\rangle =\varphi_{n}-\varphi_{n-1}+\left(1/2\right){\left\|x_{n}-x_{n-1}\right\|}^{2}+\left\langle x_{n}-x_{n-1},x_{n+1}-x_{n}\right\rangle$, it turns into

$$\varphi_{n+1}-\varphi_{n}-\beta_{n}\left(\varphi_{n}-\varphi_{n-1}\right)\leq \frac{\beta_{n}}{2}\|x_{n}-x_{n-1}{\|}^{2}+\beta_{n}\left\langle x_{n}-x_{n-1},x_{n+1}-x_{n}\right\rangle -\frac{1}{4}{\left\|x_{n+1}-x_{n}\right\|}^{2}.$$

The right-hand side of the above inequality can be rewritten as

$$\frac{\beta_{n}}{2}\|x_{n}-x_{n-1}{\|}^{2}+\beta_{n}^{2}\|x_{n}-x_{n-1}{\|}^{2}-\frac{1}{4}{\left\|x_{n+1}-x_{n}-2\beta_{n}\left(x_{n}-x_{n-1}\right)\right\|}^{2},$$

that, since $\beta_{n}\in [0,\beta [$, is less than or equal to

$$\beta_{n}\|x_{n}-x_{n-1}{\|}^{2}-\frac{1}{4}{\left\|x_{n+1}-x_{n}-2\beta_{n}\left(x_{n}-x_{n-1}\right)\right\|}^{2},$$

and, taking also in consideration that $\beta_{n}<\beta <1/9<1/8$, even to

$$\frac{9}{8}\beta_{n}\|x_{n}-x_{n-1}{\|}^{2}-\frac{1}{8}{\left\|x_{n+1}-x_{n}\right\|}^{2}.$$

Therefore, one gets

(2) $$\begin{array}{c} \varphi_{n+1}-\varphi_{n}-\beta_{n}\left(\varphi_{n}-\varphi_{n-1}\right)\leq \frac{9}{8}\beta_{n}\|x_{n}-x_{n-1}{\|}^{2}-\frac{1}{8}{\left\|x_{n+1}-x_{n}\right\|}^{2}. \end{array}$$

Denoting for all k≥1

$$\mu_{k}:=\varphi_{k}-\beta_{k}\varphi_{k-1}+\frac{9}{8}\beta_{k}{\left\|x_{k}-x_{k-1}\right\|}^{2},$$

it follows that

(3) $$\begin{array}{c} \mu_{k+1}-\mu_{k}\leq \frac{9\beta -1}{8}{\left\|x_{k+1}-x_{k}\right\|}^{2}\leq 0, \end{array}$$

thus the sequence ${\left(\mu_{k}\right)}_{k}$ is nonincreasing, as n≥1 was arbitrarily chosen. Then, for all k≥1,

$$\varphi_{k}-\beta_{k}\varphi_{k-1}\leq \mu_{k}\leq \mu_{1},$$

hence

$$\varphi_{k}\leq \beta^{k}\varphi_{0}+\frac{1-\beta^{k}}{1-\beta }\mu_{1}\leq \beta^{k}\varphi_{0}+\frac{1}{1-\beta }\mu_{1},$$

and one also gets $\|x_{k+1}-x_{k}{\|}^{2}\leq \frac{8}{9\beta -1}\left(\mu_{k+1}-\mu_{k}\right)$. Consequently,

$$\begin{array}{cc} \sum_{k=1}^{n}{\left\|x_{k+1}-x_{k}\right\|}^{2} & \leq \frac{8}{1-9\beta }\left(\mu_{1}-\mu_{n+1}\right)\leq \frac{8}{1-9\beta }\left(\mu_{1}+\beta \varphi_{n}\right)\\ & \leq \frac{8}{1-9\beta }\left(\frac{1}{1-\beta }\mu_{1}+\beta^{n+1}\varphi_{0}\right)<+\infty , \end{array}$$

in particular

(4) $$\begin{array}{c} \sum_{k=1}^{+\infty }{\left\|x_{k+1}-x_{k}\right\|}^{2}<+\infty . \end{array}$$

Using the intermediate step towards (2) and denoting $\tau_{k+1}:=x_{k+1}-x_{k}-2\beta_{k}\left(x_{k}-x_{k-1}\right)$, $\theta_{k}:=\varphi_{k}-\varphi_{k-1}$ and $\delta_{k}:=\beta_{k}{\left\|x_{k}-x_{k-1}\right\|}^{2}$ for all k≥1, one obtains

(5) $$\begin{array}{c} \theta_{k+1}-\beta_{k}\theta_{k}\leq \delta_{k}-\frac{1}{4}{\left\|\tau_{k+1}\right\|}^{2}\leq \delta_{k}\;\forall k\geq 1. \end{array}$$

Then

$${\left[\theta_{k+1}\right]}_{+}\leq \frac{1}{9}{\left[\theta_{k}\right]}_{+}+\delta_{k}\;\forall k\geq 1,$$

which yields

$${\left[\theta_{n+1}\right]}_{+}\leq \frac{1}{9^{n}}{\left[\theta_{1}\right]}_{+}+\sum_{k=0}^{n-1}\frac{\delta_{n-k}}{9^{k}}.$$

Hence

$$\sum_{k=0}^{+\infty }{\left[\theta_{k+1}\right]}_{+}\leq \frac{9}{8}\left({\left[\theta_{1}\right]}_{+}+\sum_{k=0}^{+\infty }\delta_{k}\right)$$

and, as the right-hand side of this inequality is finite due to (4), so is $\sum_{k=1}^{+\infty }{\left[\theta_{k}\right]}_{+}$, too. This yields that the sequence ${\left(w_{k}\right)}_{k}$ defined as $w_{k}=\varphi_{k}-\sum_{j=1}^{k}{\left[\theta_{j}\right]}_{+}$, for all k≥1, is bounded. Moreover, $w_{k+1}-w_{k}=\varphi_{k+1}-\varphi_{k}-{\left[\varphi_{k+1}-\varphi_{k}\right]}_{+}\leq 0$ for all k≥1, thus ${\left(w_{k}\right)}_{k}$ is convergent. Consequently, ${\left(\varphi_{k}\right)}_{k}$ is convergent. Finally, $\left(\right\|x_{k}-\tilde{x}{{\|}^{2})}_{k}$ is convergent, too, i.e. (*a*) in Lemma 1.3 with S=Ω is fulfilled.

The next step is to show that ${\left(x_{k}\right)}_{k}$ is weakly convergent. The convergence of ${\left(\varphi_{k}\right)}_{k}$ implies that ${\left(x_{k}\right)}_{k}$ is bounded, so it has weak cluster points. Let $\hat{x}\in X$ be one of them and ${\left(x_{k_{j}}\right)}_{j}$ the subsequence that converges weakly towards it as j→+∞. Then, as F+G is positively *C*-lower semicontinuous and *C*-convex, it follows that for any $z^{*}\in C^{*}\backslash \left\{0\right\}$ the function $\left\langle z^{*},\left(F+G\right)\left(\cdot \right)\right\rangle$ is lower semicontinuous and convex, thus

(6) $$\begin{array}{c} \left\langle z^{*},\left(F+G\right)\left(\hat{x}\right)\right\rangle \leq \underset{j\to +\infty }{lim inf}\left\langle z^{*},\left(F+G\right)\left(x_{k_{j}}\right)\right\rangle =\underset{k\geq 1}{\inf}\left\langle z^{*},\left(F+G\right)\left(x_{k}\right)\right\rangle , \end{array}$$

with the last equality following from the fact that the sequence ${\left(\left(F+G\right)\left(x_{k}\right)\right)}_{k}$ is by construction *C*-nonincreasing. Assuming that there exists a k≥1 such that $\left(F+G\right){\left(\hat{x}\right)}_{C}\left(F+G\right)\left(x_{k}\right)$, there exists a $\tilde{z}\in C^{*}\backslash \left\{0\right\}$ such that $\langle \tilde{z},\left(F+G\right)\left(\hat{x}\right)-\left(F+G\right)\left(x_{k}\right)\rangle >0$, which contradicts (6), consequently $\left(F+G\right)\left(\hat{x}\right){≦}_{C}\left(F+G\right)\left(x_{k}\right)$ for all k≥1, i.e. $\hat{x}\in \Omega$, therefore one can employ Lemma 1.3 with S=Ω since its hypothesis (*b*) is fulfilled as well. This guarantees then the weak convergence of ${\left(x_{k}\right)}_{k}$ to a point $\overset{¯}{x}\in \Omega$.

The proof is not complete without showing that $\overset{¯}{x}\in \mathrm{WE}(VP)$. Assuming the opposite, there would exist an $x^{\prime }\in X$ such that $\left(F+G\right)\left(x^{\prime }\right)\in \left(F+G\right)\left(\overset{¯}{x}\right)-\;\text{int}C$. This yields $x^{\prime }\in \Omega$. Since $\|z_{k}^{*}\|=1$ for all k≥1, the sequence ${\left(z_{k}^{*}\right)}_{k}$ has a weak${}^{*}$ cluster point, say ${\overset{¯}{z}}^{*}$, that is the limit of a subsequence ${\left(z_{k_{j}}^{*}\right)}_{j}$. Because $z_{k}^{*}\in C^{*}$ for all k≥1 and $C^{*}$ is weakly${}^{*}$ closed, it follows that ${\overset{¯}{z}}^{*}\in C^{*}$. Moreover, from [15, Lemma 2.2] it follows that $\langle {\overset{¯}{z}}^{*},c\rangle >0$ for any c∈intC, thus ${\overset{¯}{z}}^{*}\neq 0$. Consequently, $\langle {\overset{¯}{z}}^{*},\left(F+G\right)\left(x^{\prime }\right)-\left(F+G\right)\left(\overset{¯}{x}\right)\rangle <0$. For any j≥1 it holds then by (1) and employing the descent lemma and the $\frac{1}{L}$-cocoercivity of $\nabla (z_{k_{j}}^{*}F)$

(7) $$\begin{array}{cc} & \left\langle z_{k_{j}}^{*},\left(F+G\right)\left(x^{\prime }\right)-\left(F+G\right)\left(\overset{¯}{x}\right)\right\rangle \geq \left\langle z_{k_{j}}^{*},\left(F+G\right)\left(x^{\prime }\right)-\left(F+G\right)\left(x_{k_{j}+1}\right)\right\rangle \\ & \;\geq -L\langle x_{k_{j}+1}-x_{k_{j}}-\beta_{k_{j}}\left(x_{k_{j}}-x_{k_{j}-1}\right)+\;\frac{1}{L}\nabla \left(z_{k_{j}}^{*}F\right)\left(x_{k_{j}}\right),x^{\prime }-x_{k_{j}+1}\rangle \;\;+\left\langle z_{k_{j}}^{*},F\left(x^{\prime }\right)-F\left(x_{k_{j}+1}\right)\right\rangle \\ & \;\geq -L\|x^{\prime }-x_{k_{j}+1}\left\|(\right\|x_{k_{j}+1}-x_{k_{j}}\|+\beta_{k_{j}}\left\|x_{k_{j}}-x_{k_{j}-1}\right\|)+\left\langle \nabla \left(z_{k_{j}}^{*}F\right)\left(x_{k_{j}}\right)-\nabla \left(z_{k_{j}}^{*}F\right)\left(x^{\prime }\right),x_{k_{j}+1}-x^{\prime }\right\rangle \\ & \;\geq -L\|x^{\prime }-x_{k_{j}+1}\left\|(\right\|x_{k_{j}+1}-x_{k_{j}}\|+\beta_{k_{j}}\|x_{k_{j}}-x_{k_{j}-1}\|)-\frac{L}{4}{\left\|x_{k_{j}+1}-x_{k_{j}}\right\|}^{2}. \end{array}$$

Because of (4) $\left(\right\|x_{k}-x_{k-1}{\left\|\right)}_{k}$ converges towards 0 for k→+∞ and so does the last expression in the inequality chain (7) when j→+∞ as well. Letting *j* converge towards +∞, (7) yields $\langle {\overset{¯}{z}}^{*},\left(F+G\right)\left(x^{\prime }\right)-\left(F+G\right)\left(\overset{¯}{x}\right)\rangle \geq 0$, contradicting the inequality obtained above. Consequently, $\overset{¯}{x}\in \mathrm{WE}(VP)$.

Remark 4: The conclusion of Theorem 2.1 remains valid when *G* is taken to be *C*-lower semicontinuous in the sense of [10, Definition 2.2.14] instead of positively *C*-lower semicontinuous.

Remark 5: As can be seen in the proof of Theorem 2.1, its conclusion remains valid if the sequence ${\left(x_{n}\right)}_{n}$ generated by Algorithm 1 fulfills the condition $\sum_{k=1}^{+\infty }\beta_{k}{\left\|x_{k}-x_{k-1}\right\|}^{2}<+\infty$ (see (4), mentioned also in the literature, for instance in [1,12]), in which case ${\left(\beta_{n}\right)}_{n}$ needs not necessarily be nondecreasing and one can take β∈[0,1[. However, this dynamic condition might be more difficult to verify since it involves the generated sequence ${\left(x_{n}\right)}_{n}$, while the static hypotheses considered in this paper can simply be imposed while defining the parameters β and ${\left(\beta_{n}\right)}_{n}$, respectively.

Remark 6: In the proof of Theorem 2.1, we have employed some ideas inspired from the ones of [4, Theorem 3.1], [12, Theorem 2.1] and [1, Theorem 2.1 and Proposition 2.1]. The difficulties encountered while adapting the techniques from the mentioned statements to our framework consisted mainly of the fact that here one has to deal, as mentioned in Remark 3, at each iteration with a different optimization problem, while in [1,12] the objective function of the considered problem is not modified as the algorithm advances.

Remark 7: Different to the inertial proximal methods proposed in the literature for solving scalar optimization problems or monotone inclusions (see, for instance, [1,12]), in our approach it is not necessary to assume the existence of a solution of the considered problem, i.e. a weakly efficient solution to (*VP*), in order to prove the convergence of Algorithm 1. The role of such a hypothesis in showing the convergence of the method has been fully covered in the proof of Theorem 2.1 by the assumed *C*-completeness hypothesis. Considering the former instead of the latter, the role of Ω would be taken by WE(VP). However, then is the inclusion $\mathrm{WE}\left(VP\right)\subseteq \Omega_{n}$ for all n≥1 not guaranteed by construction and should be separately investigated. Note moreover that assuming the existence of some $\overset{¯}{x}\in \mathrm{WE}(VP)$ does not automatically deliver the corresponding scalarizing parameter ${\overset{¯}{z}}^{*}$ that exists according to Lemma 1.1, which would probably be needed in formulating the algorithm under the mentioned hypothesis.

Remark 8: Any $z^{*}\in C^{*}\backslash \left\{0\right\}$ provides a suitable scalarization functional (whose existence is guaranteed by Lemma 1.1) for the vector optimization problems in Step 3 of Algorithm 1. This endows our method with additional flexibility properties that may prove to be useful when implementing it. Moreover, even if the function

$$x\mapsto \langle z^{*},G\left(x\right)+\frac{L}{2}{∥x-x_{n}-\beta_{n}\left(x_{n}-x_{n-1}\right)+\frac{1}{L}\nabla \left(z_{n}^{*}F\right)\left(x_{n}\right)∥}^{2}e_{n}\rangle +\delta_{\Omega_{n}}\left(x\right)$$

has, because it is lower semicontinuous and strongly convex, exactly one minimum that is $x_{n+1}$, the sequence ${\left(x_{n}\right)}_{n}$ is not uniquely determined because for each choice of $z^{*}\in C^{*}\backslash \left\{0\right\}$ one deals with a different such function. Note also that the sequence ${\left(z_{n}^{*}\right)}_{n}$ can be taken even constant, situation in which the intermediate vector optimization problems differ despite having the same objective vector function because their feasible sets become smaller at each iteration. This does not mean that the vector optimization problem (*VP*) is a priori scalarized by means of a linear continuous functional, because this scalarization is applied to the intermediate vector optimization problems not to (*VP*).

Remark 9: For determining the optimal solutions of the scalarized optimization problems attached to the vector optimization problems in Step 3 of Algorithm 1 one can employ for instance a splitting type algorithm designed for finding the optimal solutions of optimization problems consisting in minimizing sums of convex functions, like the ones proposed in [16,17]. However, the processing of the functions $\delta_{\Omega_{n}}$, n≥1, may prove to be quite difficult, due to the special structure of the sets $\Omega_{n}$, n≥1. A way to go round this nuisance is, as seen later in Section 4, by employing some other approaches for solving the intermediate scalar optimization problems, for instance one based on interior point methods.

Remark 10: Similarly to [4, Section 4], it can be shown that under additional hypotheses (for instance $\exists \delta >0:\{z^{*}\in Y^{*}:\left\langle z^{*},y\right\rangle \geq \delta \|y\|\|z^{*}\|$ for all y∈C}≠∅) Algorithm 1 can deliver efficient solutions to (*VP*) instead of weakly efficient. One can also modify Algorithm 1 in order to deliver *properly efficient solutions to (*VP*) with respect to linear scalarization* that are defined as those $\overset{¯}{x}\in \;\text{dom}G$ for which there exists a $z^{*}\in C^{*0}=\{y^{*}\in C^{*}:\left\langle y^{*},y\right\rangle >0$∀y∈C\{0}} for which $\left\langle z^{*},F\left(\overset{¯}{x}\right)+G\left(\overset{¯}{x}\right)\right\rangle \leq \left\langle z^{*},F\left(x\right)+G\left(x\right)\right\rangle$ for all x∈X. For this, the interior of *C* needs not necessarily be nonempty and the sequence ${\left(e_{n}\right)}_{n}$ should lie in C\{0}, while in Steps 2 and 3 of the algorithm one should consider properly efficient instead of weakly efficient solutions to (*VP*).

Remark 11: Vector optimization problems with the ordering cones of the image spaces having empty interiors, but nonempty generalized interiors can be found in finance mathematics (see, for instance, [18,19]) and other research fields. Motivated by them, the definition of the weakly efficient solutions to (*VP*) has been extended in some recent contributions (such as [19–21]) for the case when intC=∅ by replacing this with the *quasi interior* of *C* (i.e. the set of all y∈Y such that cl(cone(V-y))=Y, where ‘cone’ denotes the *conical hull* of the corresponding set). In order to characterize these more general weakly efficient solutions to (*VP*) one can use [21, Corollary 9] instead of Lemma 1.1. The proof of the algorithm convergence statement Theorem 2.1 can be almost entirely adapted to the new framework, but, since [15, Lemma 2.2] does not hold in case intC=∅, an alternative approach for guaranteeing that ${\overset{¯}{z}}^{*}\neq 0$ is necessary. A way to do this would be by taking the sequence ${\left(z_{n}^{*}\right)}_{n}$ constant with $z_{n}^{*}={\overset{¯}{z}}^{*}\neq 0$. On the other hand, in finitely dimensional spaces so-called relatively weakly efficient solutions can be defined when *C* has an empty interior but a nonempty relative interior and characterized by means of linear scalarization (cf. [20]), while ${\overset{¯}{z}}^{*}\neq 0$ because of the coincidence of the corresponding weak and strong topologies.

One can simplify Algorithm 1 in order to become a ‘pure’ (i.e. non-inertial) forward–backward method, as follows.


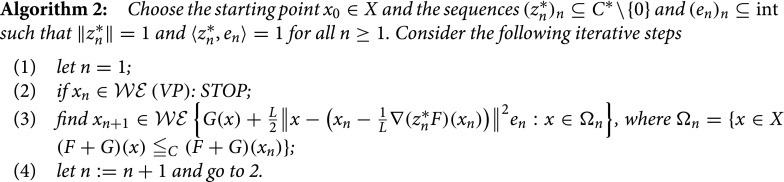


This algorithm is interesting not only per se, but also because one can derive rates for its convergence, by employing some ideas from [13], when the sequence ${\left(z_{n}^{*}\right)}_{n}$ is constant. In order to prove the corresponding statement, an additional result is necessary (following [13, Lemma 2.3]).

Lemma 2.1: Let *F* be *C*-convex and *G* be *C*-convex and positively *C*-lower semicontinuous and denote, for fixed $z^{*}\in C^{*}$, e∈intC with $\langle z^{*},e\rangle =1$ and y∈X,

$$z_{y}:=\underset{x\in \Omega_{y}}{\;\text{argmin}}\langle z^{*},G\left(x\right)+\frac{L}{2}{∥x-y+\frac{1}{L}\nabla \left(z^{*}F\right)\left(y\right)∥}^{2}e\rangle ,$$

where $\Omega_{y}=\left\{x\in X:\left(F+G\right)\left(x\right){≦}_{C}\left(F+G\right)\left(y\right)\right\}$. One has

(8) $$\begin{array}{c} \left\langle z^{*},F\left(x\right)+G\left(x\right)-F\left(z_{y}\right)-G\left(z_{y}\right)\right\rangle +\delta_{\Omega_{y}}\left(x\right)\geq \frac{L}{2}{\left\|z_{y}-y\right\|}^{2}+L\left\langle z_{y}-y,y-x\right\rangle \;\forall x\in X. \end{array}$$

**Proof** For a y∈X, by the definition of $z_{y}$ one gets, taking into consideration the continuity of the norm (like in the proof of Theorem 2.1) and that $z_{y}\in \Omega_{y}$,

$$\left\langle z^{*},G\left(x\right)-G\left(z_{y}\right)\right\rangle +\delta_{\Omega_{y}}\left(x\right)\geq \left\langle \nabla \left(z^{*}F\right)\left(y\right)+L\left(z_{y}-y\right),z_{y}-x\right\rangle \;\forall x\in X.$$

Using also that $\left\langle z^{*},F\left(x\right)-F\left(y\right)\right\rangle \geq \left\langle \nabla \left(z^{*}F\right)\left(y\right),x-y\right\rangle$ for all x∈X, one gets

$$\left\langle z^{*},F\left(x\right)+G\left(x\right)-F\left(y\right)-G\left(z_{y}\right)\right\rangle +\delta_{\Omega_{y}}\left(x\right)\geq \left\langle \nabla \left(z^{*}F\right)\left(y\right)-L\left(x-z_{y}\right),z_{y}-y\right\rangle \;\forall x\in X.$$

Employing the descent lemma, the above inequality yields

$$\left\langle z^{*},F\left(x\right)+G\left(x\right)-F\left(z_{y}\right)-G\left(z_{y}\right)\right\rangle +\delta_{\Omega_{y}}\left(x\right)\geq -\frac{L}{2}{\left\|z_{y}-y\right\|}^{2}-L\left\langle x-z_{y},z_{y}-y\right\rangle \;\forall x\in X,$$

and the right-hand side can be rewritten in order to deliver (8).

Theorem 2.2: Let *F* be *C*-convex and *G* be *C*-convex and positively *C*-lower semicontinuous and assume that $\left(F+G\right)\left(X\right)\cap (F\left(x_{0}\right)+G\left(x_{0}\right)-C)$ is *C*-complete. Consider the sequence ${\left(x_{n}\right)}_{n}$ generated by Algorithm 2, where one takes $z_{n}^{*}=z^{*}\in C^{*}\backslash \left\{0\right\}$, n≥1. Then for any n≥1 and $\tilde{x}\in \Omega$ one has

(9) $$\begin{array}{c} \left\langle z^{*},F\left(x_{n}\right)+G\left(x_{n}\right)-F\left(\tilde{x}\right)-G\left(\tilde{x}\right)\right\rangle \leq \frac{L\|\tilde{x}-x_{0}{\|}^{2}}{2n}. \end{array}$$

**Proof** In order to prove the statement, one can follow the steps from the proof of [13, Theorem 3.1] by employing twice Lemma 2.1 for the functions $\left(z^{*}F\right)$ and $\left(z^{*}G\right)$, respectively, first for $x=\tilde{x}$ and $y=x_{n}$, then for $x=y=x_{n}$ (for a fixed n≥1), taking also into consideration that $\tilde{x},x_{n}\in \Omega_{n}$.

Remark 12: Note that the assertion of Theorem 2.2 is actually valid for all $\tilde{x}\in \Omega$, not only for the weakly efficient solution to (*VP*) obtained from the convergence statement Theorem 2.1. Moreover, when taking the sequence ${\left(z_{n}^{*}\right)}_{n}$ constant it is no longer necessary to take $\|z_{n}^{*}\|=1$ for all n≥1. However, the constant $z^{*}$ cannot be taken arbitrarily large (with respect to the ordering cone $C^{*}$) because it has to fulfill $\langle z^{*},e_{n}\rangle =1$ for all n≥1. Moreover, in this case one can consider the more general framework discussed in Remark 11. Without assuming that the sequence ${\left(z_{n}^{*}\right)}_{n}$ is constant, in order to show instead of (9) in a similar manner to the proof of Theorem 2.2 that

$$\left\langle z_{n}^{*},F\left(x_{n}\right)+G\left(x_{n}\right)-F\left(\tilde{x}\right)-G\left(\tilde{x}\right)\right\rangle \leq \frac{L\|\tilde{x}-x_{0}{\|}^{2}}{2n}\;\forall n\geq 1\;\forall \tilde{x}\in \Omega ,$$

one needs additional assumptions that guarantee certain monotonicity properties for ${\left(z_{n}^{*}\right)}_{n}$, for instance

$$\left\langle z_{n}^{*}-z_{n+1}^{*},F\left(x_{n+1}\right)+G\left(x_{n+1}\right)\right\rangle \geq 0\geq \left\langle z_{n}^{*}-z_{n+1}^{*},F\left(\tilde{x}\right)+G\left(\tilde{x}\right)\right\rangle .$$

Remark 13: For implementation purposes, one can provide an inexact version of Algorithm 1 as well, where Step 3 is replaced by

3’ find $x_{n+1}\in X$ such that $0\in \partial_{\varepsilon_{n}}(\langle z_{n}^{*},G\left(\cdot \right)+\frac{L}{2}\|\cdot -x_{n}-\beta_{n}\left(x_{n}-x_{n-1}\right)+\frac{1}{L}\nabla \left(z_{n}^{*}F\right)\left(x_{n}\right){\|}^{2}e_{n}\rangle +\delta_{\Omega_{n}}\left(\cdot \right))\left(x_{n+1}\right)$,

where the additional sequence of tolerable nonnegative errors ${\left(\varepsilon_{n}\right)}_{n}$ fulfills some hypotheses, such as the ones considered in [4] or those from [2,12]. Employing the later, i.e. $\sum_{n\geq 1}\varepsilon_{n}<+\infty$, the converge statement obtained by correspondingly modifying Theorem 2.1 remains valid, only some minor adjustments in the proof (for instance one takes $\delta_{k}=\beta_{k}{\left\|x_{k}-x_{k-1}\right\|}^{2}+\varepsilon_{k}$ for k≥1) being necessary.

## 3. Alternative inertial forward–backward algorithm

One can modify Algorithm 1 into another inertial forward–backward method, as follows.


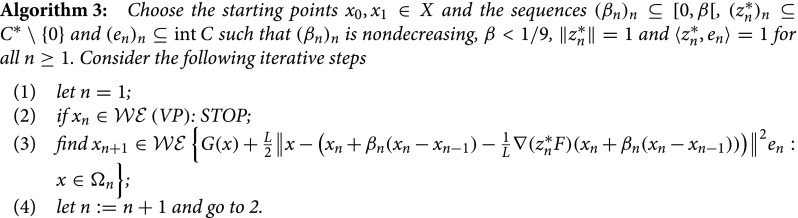

Remark 14: The difference between Algorithm 1 and Algorithm 3 resides in the point where the value of $\nabla (z_{n}^{*}F)$ is calculated, $x_{n}$ vs. $x_{n}+\beta_{n}\left(x_{n}-x_{n-1}\right)$. Thus, the comments from Remark 2 for F≡0 remain valid for Algorithm 3 as well. On the other hand, when Y=R and $C=R_{+}$ (i.e. in the scalar case), Algorithm 3 becomes a more general version of the celebrated FISTA method from [13], that can be recovered by taking $\beta_{n}=\left(t_{n}-1\right)/t_{n+1}$, where $t_{n+1}=\left(1+\sqrt{1+4t_{n}^{2}}\right)/2$, n≥1, with $t_{1}=1$, and restricting the framework to finitely dimensional spaces.

The convergence of the sequence ${\left(x_{n}\right)}_{n}$ generated by Algorithm 3 can be investigated in an analogous manner to Theorem 2.1, hence the proof of the next statement contains only what is different compared to the mentioned statement.

Theorem 3.1: Let *F* be *C*-convex and *G* be *C*-convex and positively *C*-lower semicontinuous and assume that $\left(F+G\right)\left(X\right)\cap (F\left(x_{0}\right)+G\left(x_{0}\right)-C)$ is *C*-complete. If Algorithm 3 does not stop in finitely many steps, then any sequence ${\left(x_{n}\right)}_{n}$ generated by it converges weakly towards a weakly efficient solution to (*VP*).

**Proof** Let be n≥1. Like in the proof of Theorem 2.1 the algorithm is well defined and one gets

$$\begin{array}{cc} & \left\langle L\left(x_{n+1}-x_{n}-\beta_{n}\left(x_{n}-x_{n-1}\right)\right)+\nabla \left(z_{n}^{*}F\right)\left(x_{n}+\beta_{n}\left(x_{n}-x_{n-1}\right)\right)-\nabla \left(z_{n}^{*}F\right)\left(\tilde{x}\right),x_{n+1}-\tilde{x}\right\rangle \\ & \;\leq \frac{L}{2}{\left\|\tilde{x}-x_{n+1}\right\|}^{2}, \end{array}$$

which, by employing the $\frac{1}{L}$-cocoercivity of $\nabla (z_{n}^{*}F)$, yields

$$\left\langle x_{n+1}-x_{n}-\beta_{n}\left(x_{n}-x_{n-1}\right),x_{n+1}-\tilde{x}\right\rangle -\frac{1}{2}\|\tilde{x}-x_{n+1}{\|}^{2}-\frac{1}{4}{\left\|x_{n+1}-x_{n}-\beta_{n}\left(x_{n}-x_{n-1}\right)\right\|}^{2}\leq 0.$$

Denoting $\varphi_{k}=\left(1/2\right){\left\|x_{k}-\tilde{x}\right\|}^{2}$, for k≥1, one gets, like in the proof of Theorem 2.1,

$$\begin{array}{cc} \varphi_{n+1}-\varphi_{n}-\beta_{n}\left(\varphi_{n}-\varphi_{n-1}\right) & \leq \frac{\beta_{n}}{2}{\left\|x_{n}-x_{n-1}\right\|}^{2}+\beta_{n}\left\langle x_{n}-x_{n-1},x_{n+1}-x_{n}\right\rangle \\ & \;-\frac{1}{4}{\left\|x_{n+1}-x_{n}-\beta_{n}\left(x_{n}-x_{n-1}\right)\right\|}^{2}. \end{array}$$

The right-hand side of the above inequality can be rewritten, taking into consideration that $\|x_{n+1}-x_{n}-\beta_{n}\left(x_{n}-x_{n-1}\right){\|}^{2}=\|x_{n+1}-x_{n}{\|}^{2}+\beta_{k}^{2}{\left\|x_{n}-x_{n-1}\right\|}^{2}-2\beta_{n}\left\langle x_{n+1}-x_{n},x_{n}-x_{n-1}\right\rangle$, as

$$\frac{\beta_{n}}{2}\|x_{n}-x_{n-1}{\|}^{2}-\frac{\beta_{n}^{2}}{4}\|x_{n}-x_{n-1}{\|}^{2}-\frac{1}{4}{\left\|x_{n+1}-x_{n}\right\|}^{2}+\frac{3}{2}\beta_{n}\left\langle x_{n+1}-x_{n},x_{n}-x_{n-1}\right\rangle ,$$

being further equal to

$$\left(\frac{\beta_{n}}{2}+2\beta_{n}^{2}\right)\|x_{n}-x_{n-1}{\|}^{2}-\frac{1}{4}{\left\|x_{n+1}-x_{n}-3\beta_{n}\left(x_{n}-x_{n-1}\right)\right\|}^{2}.$$

Therefore, $\varphi_{n+1}-\varphi_{n}-\beta_{n}\left(\varphi_{n}-\varphi_{n-1}\right)$ is less than or equal to

$$\frac{\beta_{n}}{2}\left(1+2\beta_{n}\right)\|x_{n}-x_{n-1}{\|}^{2}-\frac{1}{4}{\left\|x_{n+1}-x_{n}-3\beta_{n}\left(x_{n}-x_{n-1}\right)\right\|}^{2}.$$

From

$$-\frac{1}{4}\|x_{n+1}-x_{n}-3\beta_{n}\left(x_{n}-x_{n-1}\right){\|}^{2}\leq \frac{9}{4}\beta_{n}^{2}\|x_{n}-x_{n-1}{\|}^{2}-\frac{1}{8}{\left\|x_{n+1}-x_{n}\right\|}^{2},$$

one gets

$$\varphi_{n+1}-\varphi_{n}-\beta_{n}\left(\varphi_{n}-\varphi_{n-1}\right)\leq \beta_{n}(\frac{1}{2}+\frac{17}{4}\beta_{n})\|x_{n}-x_{n-1}{\|}^{2}-\frac{1}{8}{\left\|x_{n+1}-x_{n}\right\|}^{2},$$

that, since $\beta_{n}<1/8$, yields (2).

The rest of the proof follows analogously to the one of Theorem 2.1.

Remark 15: Making use of Lemma 2.1, one can provide, following [13, Theorem 4.4], a convergence rate statement for Algorithm 3 for a special choice of the parameters $\beta_{n}$, n≥1, and when the sequence ${\left(z_{n}^{*}\right)}_{n}$ is constant, that improves the assertion in the non-inertial case from Theorem 2.2.

As discussed for instance also in [4], one can consider in the presented algorithms a stopping rule that is easier to check than the original one. The following statement shows that if three consecutive iterations of the sequence ${\left(x_{n}\right)}_{n}$ generated by the inertial type algorithms we proposed coincide, they represent a weakly efficient solution to (*VP*), i.e. Step 2 of Algorithm 1 or Algorithm 3 can be replaced with

2’ if $x_{n+1}=x_{n}=x_{n-1}$: STOP.

Proposition 3.1: Let *F* and *G* be *C*-convex and consider a sequence ${\left(x_{k}\right)}_{k}$ generated by Algorithm 1. If for some n≥1 one has

$$x_{n-1}=x_{n}\in \underset{x\in \Omega_{n}}{\;\text{argmin}}\langle z_{n}^{*},G\left(x\right)+\frac{L}{2}{∥x-(x_{n}+\beta_{n}\left(x_{n}-x_{n-1}\right)-\frac{1}{L}\nabla \left(z_{n}^{*}F\right)\left(x_{n}\right))∥}^{2}e_{n}\rangle ,$$

then $x_{n}\in \mathrm{WE}\left(VP\right)$.

**Proof** Assuming that $x_{n}\notin \mathrm{WE}\left(VP\right)$, there exist $\tilde{x}\in X$ and c∈intC such that $F\left(\tilde{x}\right)+G\left(\tilde{x}\right)=F\left(x_{n}\right)+G\left(x_{n}\right)-c$. Then $\tilde{x}\in \Omega_{n}$. Denoting, for t∈[0,1[, $x_{t}:=tx_{n}+\left(1-t\right)\tilde{x}$, the *C*-convexity of F+G yields $F\left(x_{t}\right)+G\left(x_{t}\right)\leq_{C}F\left(x_{n}\right)+G\left(x_{n}\right)+\left(t-1\right)c$, therefore $x_{t}\in \Omega_{n}$ as well.

Since

$$x_{n}\in \underset{x\in \Omega_{n}}{\;\text{argmin}}\langle z_{n}^{*},G\left(x\right)+\frac{L}{2}{∥x-(x_{n}+\beta_{n}\left(x_{n}-x_{n-1}\right)-\frac{1}{L}\nabla \left(z_{n}^{*}F\right)\left(x_{n}\right))∥}^{2}e_{n}\rangle ,$$

one gets then

$$\begin{array}{cc} 0 & \leq \left\langle z_{n}^{*},G\left(x_{t}\right)-G\left(x_{n}\right)\right\rangle +\frac{L}{2}{∥x_{t}-\left(x_{n}+\beta_{n}\left(x_{n}-x_{n-1}\right)-\frac{1}{L}\nabla \left(z_{n}^{*}F\right)\left(x_{n}\right)\right)∥}^{2}\\ & \;-\frac{L}{2}{∥x_{n}-\left(x_{n}+\beta_{n}\left(x_{n}-x_{n-1}\right)-\frac{1}{L}\nabla \left(z_{n}^{*}F\right)\left(x_{n}\right)\right)∥}^{2}, \end{array}$$

which, employing the definition of $x_{t}$ and the hypothesis $x_{n-1}=x_{n}$, yields

(10) $$\begin{array}{c} 0\leq \left\langle z_{n}^{*},G\left(x_{t}\right)-G\left(x_{n}\right)\right\rangle +\frac{L}{2}{∥\left(1-t\right)\left(\tilde{x}-x_{n}\right)+\frac{1}{L}\nabla \left(z_{n}^{*}F\right)\left(x_{n}\right))∥}^{2}-\frac{1}{2L}\|\nabla \left(z_{n}^{*}F\right)\left(x_{n}\right){)\|}^{2}. \end{array}$$

As *G* is *C*-convex, one has $\left\langle z_{n}^{*},G\left(x_{t}\right)-G\left(x_{n}\right)\right\rangle \leq \left(1-t\right)\left\langle z_{n}^{*},G\left(\tilde{x}\right)-G\left(x_{n}\right)\right\rangle$, while

$$\begin{array}{cc} {∥\left(1-t\right)\left(\tilde{x}-x_{n}\right)+\frac{1}{L}\nabla \left(z_{n}^{*}F\right)\left(x_{n}\right))∥}^{2} & ={\left(1-t\right)}^{2}\|\left(\tilde{x}-x_{n}\right){\|}^{2}+\frac{1}{L^{2}}\|\nabla \left(z_{n}^{*}F\right)\left(x_{n}\right){)\|}^{2}\\ & \;+\left(1-t\right)\frac{2}{L}\left\langle \tilde{x}-x_{n},\nabla \left(z_{n}^{*}F\right)\left(x_{n}\right)\right\rangle , \end{array}$$

consequently, (10) yields

$$0\leq \left(1-t\right)\left\langle z_{n}^{*},G\left(\tilde{x}\right)-G\left(x_{n}\right)\right\rangle +\frac{L}{2}{\left(1-t\right)}^{2}{\left\|\left(\tilde{x}-x_{n}\right)\right\|}^{2}+\left(1-t\right)\left\langle \tilde{x}-x_{n},\nabla \left(z_{n}^{*}F\right)\left(x_{n}\right)\right\rangle .$$

Dividing with 1-t and using the convexity of $\left(z_{n}^{*}F\right)$, one gets

$$0\leq \left\langle z_{n}^{*},G\left(\tilde{x}\right)-G\left(x_{n}\right)\right\rangle +\frac{L}{2}\left(1-t\right){\left\|\left(\tilde{x}-x_{n}\right)\right\|}^{2}+\left\langle z_{n}^{*},F\left(\tilde{x}\right)-F\left(x_{n}\right)\right\rangle ,$$

followed by $\left\langle z_{n}^{*},c\right\rangle \leq \frac{L}{2}\left(1-t\right){\left\|\left(\tilde{x}-x_{n}\right)\right\|}^{2}$.

Letting *t* tend towards 1 and using that $z_{n}^{*}\in C^{*}\backslash \left\{0\right\}$ and c∈intC, the last inequality yields $0<\langle z_{n}^{*},c\rangle \leq 0$, which is a contradiction, consequently, $x_{n}\in \mathrm{WE}\left(VP\right)$.

Remark 16: Note that $x_{n-1}=x_{n}$ does not necessarily imply that $x_{n+1}$ coincides with them, too, but the fact that it depends only on $x_{n}$ and not on $x_{n-1}$. This can prove to be useful when starting the algorithm because one can begin with $x_{0}=x_{1}$ without affecting the convergence of the method.

## 4. Numerical experiments

In order to verify the proposed methods, we present in the following an example where a multiobjective optimization problem is solved by implementing Algorithm 1 in Matlab (version 9.0.0.341360/R2016a) on a Windows 7-PC with an Intel Core i5 processor with 3.40 GHz and 8 GB of RAM.

Consider the vector optimization problem

$$\left(EP\right)\;\;\underset{\begin{array}{c} x=\left(x_{1},\cdots ,x_{d}\right)\in R_{+}^{d},\\ \sum_{i=1}^{d}x_{i}=1 \end{array}}{\;\text{Min}}\left(\begin{array}{c} -x^{⊤}u\\ x^{⊤}Vx \end{array}\right),$$

where $u\in R^{d}$ and $V\in R^{d\times d}$ is a symmetric positive semidefinite matrix. Such problems can be found, for instance, in portfolio optimization, where *x* can be interpreted as the portfolio vector for *d* given assets having the proportions of the assets in the whole portfolio as components where the short sales are excluded, the first component of the objective vector function represents the negative of the expected return (that is to be maximized, therefore minimized with a leading minus), while the second is the variance of the portfolio, expressed by a quadratic function involving a symmetric positive semidefinite variance–covariance matrix $V\in R^{d\times d}$, that quantifies the risk associated to the considered portfolio and should be concomitantly minimized.

The vector optimization problem (*EP*) can be recast as a special case of (*VP*) by taking $X=R^{d}$, $Y=R^{2}$, $C=R_{+}^{2}$, $F\left(x\right)={\left(-x^{⊤}u,x^{⊤}Vx\right)}^{⊤}$ and $G\left(x\right)={\left(\delta_{R_{+}^{d}\cap T}\left(x\right),\delta_{R_{+}^{d}\cap T}\left(x\right)\right)}^{⊤}$, where $T=\{x=\left(x_{1},\cdots ,x_{d}\right)\in R^{d}:\sum_{i=1}^{d}x_{i}=1\}$. Note that *F* is proper, $R_{+}^{2}$-convex and Fréchet differentiable and has a Lipschitz continuous gradient $\nabla F\left(x\right)={\left(u,Vx\right)}^{⊤}$ and *G* is proper, $R_{+}^{2}$-convex and positively $R_{+}^{2}$-lower semicontinuous.

For the concrete implementation of the method, we use the real data considered in [22] that contains five stocks IBM, Microsoft, Apple, Quest Diagnostics and Bank of America, whose expected return and variance in the portfolio were calculated based on historical stock price and dividend payment from 1 February 2002 to 1 February 2007. Consider thus the problem (*EP*) with d=5, $u={\left(0.4,0.513,4.085,1.006,1.236\right)}^{⊤}$ and

$$V=\left(\begin{array}{ccccc} 0.006461 & 0.002983 & 0.00235487 & 0.00235487 & 0.00096889\\ 0.002983 & 0.0039 & 0.00095937 & -0.0001987 & 0.00063459\\ 0.002355 & 0.000959 & 0.01267778 & 0.00135712 & 0.00134481\\ 0.002355 & -0.0002 & 0.00135712 & 0.00559836 & 0.00041942\\ 0.000969 & 0.000635 & 0.00134481 & 0.00041942 & 0.0016229 \end{array}\right).$$

Take moreover $z_{n}^{*}={\left(1/\sqrt{2},1/\sqrt{2}\right)}^{⊤}$ and $e_{n}={\left(1,1\right)}^{⊤}$ for all n≥1, and as the starting points of the algorithm $x_{0}={\left(0.25,0.25,0,0.25,0.25\right)}^{⊤}$ and $x_{1}={\left(0.15,0.25,0.25,0.2,0.15\right)}^{⊤}$. Note that this choice of the scalarization function guarantees, in the light of Remark 10, that the iteratively generated sequence actually converges towards a properly efficient solution to (*EP*), that is, consequently, also efficient. In order to obtain only weakly efficient solutions to (*EP*), that are, as pointed by an anonymous reviewer, not quite relevant for the problem in discussion, one could take some components of the $z_{n}^{*}$ to be 0. The intermediate problems scalarized with the corresponding $z_{n}^{*}$’s become

$$\left(SP_{n}\right)\;\;\underset{\begin{array}{c} x=\left(x^{1},x^{2},x^{3},x^{4},x^{5}\right)\in R_{+}^{5},\\ x^{1}+x^{2}+x^{3}+x^{4}+x^{5}=1,\\ u^{⊤}\left(x-x_{n}\right)\leq 0,\\ x^{⊤}Vx-x_{n}^{⊤}Vx_{n}\leq 0 \end{array}}{\inf}\frac{1}{2}{\left\|x-x_{n}-\beta_{n}\left(x_{n}-x_{n-1}\right)+\frac{1}{\sqrt{2}}\left(u+Vx_{n}\right)\right\|}^{2},\;n\geq 1.$$

For computational reasons, we consider an inexact version of the stopping rule $2^{\prime }$, namely that $\|x_{n+1}-x_{n}\left\|\leq \varepsilon =0.00001\geq \right\|x_{n}-x_{n-1}\|$. The intermediate scalar problems $\left(SP_{n}\right)$ are solved using the Matlab function fmincon, the existing proximal-point methods being not employable because of the complicated constraint sets.

In the following tables, we present some of the achieved computational results. Taking the sequence ${\left(\beta_{n}\right)}_{n}$ constant, the program delivers the approximate properly efficient solution $\overset{¯}{x}={\left(0.00000015603,0.0718,0.3189,0.1317,0.4777\right)}^{⊤}$ to (*EP*) after 15.517354 seconds and 281 iterations when $\beta_{n}=1/10$, n≥1. Although there is no certain rule, one can notice that when the value of $\beta_{n}$ decreases the elapsed time and the number of iterations tend to increase. However, when the inertial step is omitted, i.e. $\beta_{n}=0$ for all n≥1, and the method becomes a ‘pure’ forward–backward one, the algorithm needs 33.288016 seconds and 625 iterations until the stopping rule is activated. In all these cases fmincon delivers approximate global optimal solutions for the intermediate problems. On the other hand, when the sequence ${\left(\beta_{n}\right)}_{n}$ is nondecreasing but not constant, fmincon delivers for the intermediate problems mostly approximate local optimal solutions, however the elapsed time and the number of iterations decrease dramatically, less than a second being necessary to deliver the approximate properly efficient solution $\overset{¯}{x}={\left(0.000000046008,0.00000072695,0.3050,0.0878,0.6072\right)}^{⊤}$ to (*EP*) after 13 iterations when $\beta_{n}=1/30-1/\left(n+30\right)$, n≥1.

**Table:** 

$\beta_{n}$	Iterations	Time (s)
0	625	33.288016
$\frac{1}{10}$	281	15.517354
$\frac{1}{11}$	579	31.590278
$\frac{1}{12}$	707	38.557769
$\frac{1}{15}$	603	32.456625
$\frac{1}{20}$	507	28.100981
$\frac{1}{25}$	507	27.316595
$\frac{1}{30}$	571	30.952835
$\frac{1}{50}$	749	40.752453
$\frac{1}{100}$	813	43.925126
$\frac{1}{300}$	783	39.746160
$\frac{1}{500}$	1083	60.644596
$\frac{1}{10}-\frac{1}{10n}$	13	1.080089
$\frac{1}{10}-\frac{1}{n+10}$	117	11.592547
$\frac{1}{11}-\frac{1}{n+11}$	117	11.784833
$\frac{1}{15}-\frac{1}{n+15}$	117	11.692819
$\frac{1}{20}-\frac{1}{n+20}$	15	1.721906
$\frac{1}{25}-\frac{1}{n+25}$	13	1.038344
$\frac{1}{30}-\frac{1}{n+30}$	13	0.988701
$\frac{1}{50}-\frac{1}{n+50}$	15	1.245741
$\frac{1}{100}-\frac{1}{n+100}$	15	1.989177
$\frac{1}{300}-\frac{1}{n+300}$	15	1.960903
$\frac{1}{500}-\frac{1}{n+500}$	15	1.975370

## 5. Conclusions and further research

In this paper, we propose two forward–backward proximal point type algorithms with inertial/memory effects for determining weakly efficient solutions to a vector optimization problem consisting in vector-minimizing with respect to a given closed convex pointed cone the sum of a proper cone-convex vector function with a cone-convex differentiable one, the first ones with these characteristics in the literature, to the best of our knowledge. Among the ideas we consider for future work in this research direction we mention first the identification of possible ways to avoid using the constraint sets $\Omega_{n}$, n≥1, without losing the convergence of the method. Likewise, we are also interested in finding alternative hypotheses to the *C*-completeness of $\left(F+G\right)\left(X\right)\cap (F\left(x_{0}\right)+G\left(x_{0}\right)-C)$ that are weaker than the ones mentioned in [4, Remark 3] as well as in providing convergence rates for our algorithm in more general frameworks than the one we give in the non-inertial case when the scalarizing sequence is constant. Moreover, we plan to investigate some ways to modify the proposed algorithms in order to encompass as a special case also the projected gradient method proposed in [8] for vector-minimizing a smooth cone-convex vector function. Extending our investigations by employing Bregman type distances instead of the classical one like in [9] is another idea worth exploring.
